# Netrin-1 suppresses the MEK/ERK pathway and ITGB4 in pancreatic cancer

**DOI:** 10.18632/oncotarget.8348

**Published:** 2016-03-25

**Authors:** Xi-Zhou An, Zhi-Guo Zhao, Yu-Xuan Luo, Ran Zhang, Xiao-Qiang Tang, De-Long Hao, Xiang Zhao, Xiang Lv, De-Pei Liu

**Affiliations:** ^1^ State Key Laboratory of Medical Molecular Biology, Department of Biochemistry and Molecular Biology, Institute of Basic Medical Sciences, Chinese Academy of Medical Sciences and Peking Union Medical College, Beijing 100005, P.R. China

**Keywords:** netrin-1, pancreatic ductal adenocarcinoma, PP2A, MEK/ERK, integrin-beta4

## Abstract

The axon guidance factor netrin-1 promotes tumorigenesis in multiple types of cancers, particularly at their advanced stages. Here, we investigate whether netrin-1 is involved in the *in vivo* growth of pancreatic adenocarcinoma. We show that netrin-1 is significantly under-expressed in stage-I/II pancreatic ductal adenocarcinoma (PDAC). Netrin-1 over-expression effectively arrests the growth of xenografted PDAC cells without decreasing cell proliferation or increasing apoptosis in two-dimensional cultures *in vitro*. Integrin-beta4 (ITGB4) expression is significantly reduced, and ITGB4-knockdown mimics the tumor-suppressive effect of netrin-1, implying that ITGB4 is a main target of netrin-1 in constraining PDAC. We further show that netrin-1 signals to UNC5B/FAK to stimulate nitric oxide production, which promotes PP2A-mediated inhibition of the MEK/ERK pathway and decreases phosphorylated-c-Jun recruitment to the ITGB4 promoter. Our findings suggest that netrin-1 can suppress the growth of PDAC and provide a mechanistic insight into this suppression.

## INTRODUCTION

Pancreatic ductal adenocarcinoma (PDAC) is one of the most lethal human cancers worldwide and is notorious for its invasion of surrounding tissues, early metastasis and ability to evade early diagnosis [1]. The occurrence and death rate of PDAC remain largely unchanged after decades of studies on this devastating malignancy [2]. The complex crosstalk of PDAC cells with the surrounding tumor microenvironment is an attractive field of study and suggests potential targets for therapeutic intervention [3].

Netrin-1 is a canonical extracellular axon guidance cue [4, 5] and also regulates angiogenesis and epithelial branching events. This protein is also involved in tumorigenesis as a survival factor by inhibiting the proapoptotic effect of its dependent receptors, the DCC and UNC5 family members [6–9]. Increased netrin-1 expression has been observed in many types of advanced cancers [10]. The impact of netrin-1 on tumor progression is further complicated by its roles in angiogenesis, as both attractive and inhibitory effects on the extension of blood vessels have been documented [11–13]. Netrin-1 is also expressed in the pancreas and modulates fetal pancreatic branching morphogenesis by affecting the adhesion and migration of duct progenitor cells [14]. Up-regulated netrin-1 expression has been observed in a chick chorioallantoic membrane model of PDAC cell invasion [15] and in some human PDAC samples that are associated with worse prognosis [16], suggesting a pro-tumorigenic role of netrin-1 in advanced PDAC. Whether and how netrin-1 affects the *in vivo* growth of PDAC remain unknown.

The integrin family of receptors represents the main group of proteins that mediate cell adhesion to the ECM and transmit extracellular signals. Interestingly, integrin α6β4 has been indicated as a receptor for netrin-1 during the branching morphogenesis of the pancreas duct in the fetal pancreas and mediates the adhesion and migration of pancreatic epithelial cells [14]. Accumulating evidence has indicated that integrin α6β4 is involved in the development of invasive and metastatic adenocarcinomas [17]. Shaw and colleagues have demonstrated that integrin β4 can activate PI3K to induce the invasion of the MDA-MB-435 breast carcinoma cell line [18]. Elevated α6β4 expression has been noted in many types of carcinomas [19]. Specifically, integrin α6β4 over-expression and translocation from the basement membrane to the inner-space of ductal epithelial cells was identified as a marker for the early-stage of pancreatic adenocarcinoma [20].

In the present study, we found that netrin-1 was expressed in the acini of normal pancreatic tissue and that this expression was significantly reduced in early-stage PDAC samples. Netrin-1 over-expression notably inhibited the tumorigenicity of PDAC cells in xenograft models and in a Matrigel matrix. Further investigation showed that netrin-1 decreased cell adhesion to ECM components but did not affect the *in vitro* proliferation or apoptosis of PDAC cells in two-dimensional (2D) cultures. Integrin β4 expression was reduced following netrin-1 stimulation and mediated, at least in part, the observed tumor-inhibitory effect of netrin-1. The signaling pathway from netrin-1 to integrin β4 requires its receptor, UNC5b, and the activation of FAK, which in turn stimulates nitric oxide production, mediates PP2A-induced inhibition of the MEK/ERK/c-Jun pathway, and decreases the recruitment of phosphorylated c-Jun to the integrin β4 promoter.

## RESULTS

### Netrin-1 expression is decreased in early-stage PDAC samples

**Figure 1:**
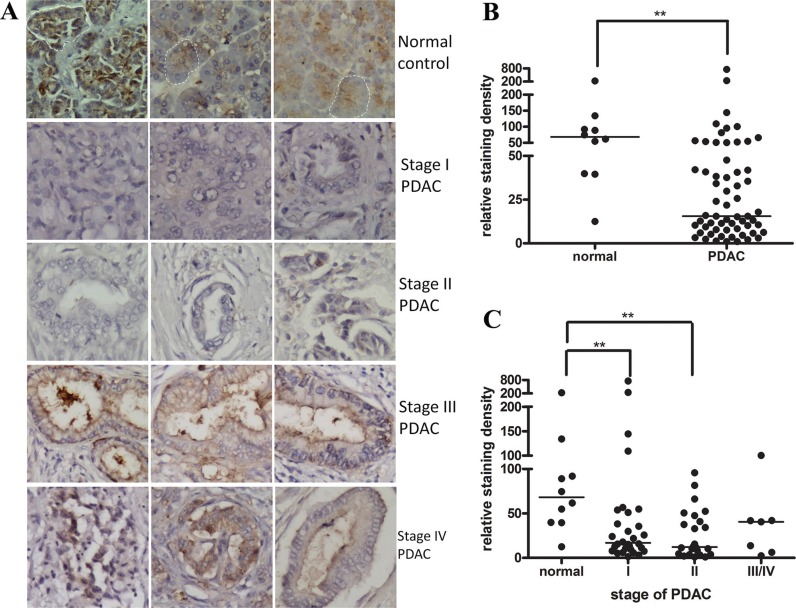
Netrin-1 expression is decreased in early-stage PDAC samples (**A**) Netrin-1 immunohistochemical staining (ab122903, Abcam) in normal pancreatic tissue and stage I–IV pancreatic ductal adenocarcinoma (PDAC). Three representative graphs of each stage are shown (200×). The dashed circles show representative acini that are positive for netrin-1 staining in the normal pancreatic tissue. It is difficult to observe the acinar cells and netrin-1 staining in the stage I and II PDAC samples. The ductal expression of netrin-1 becomes evident in stage III and IV PDACs. (**B**) Statistical analyses of the netrin-1 expression level in the normal pancreatic tissues (*N* = 10) and the total PDAC tumors (*N* = 61) on the immunostained tissue array (**P < 0.01). The line refers to the group median. (**C**) Stage-specific analysis of netrin-1 expression in the PDAC samples (*N* = 30 for stage I, *N* = 24 for stage II, and *N* = 7 for stages III/IV) compared with that in the normal pancreas tissues (*N* = 10) on the immunostained tissue array (*P < 0.05, **P < 0.01). The line refers to the group median.

We first characterized the netrin-1 expression pattern during PDAC progression using a human pancreatic cancer tissue array containing all stages of ductal adenocarcinoma and normal pancreatic tissue. Immunohistochemical staining with an anti-netrin-1 antibody (ab122903) showed a clear netrin-1 signal in the exocrine portion of the normal pancreas, predominantly in the acini cells (Figure 1A). The netrin-1 signal was obviously decreased in the stage-I/II PDAC samples, accompanied by an acute disappearance of the acini cells (Figure 1A). Conversely, significant ductal expression of netrin-1 was observed in the stage-III/IV PDAC samples (Figure 1A), consistent with a previous report [15]. Overall, the analyses showed that netrin-1 expression was reduced in the PDAC samples compared with the normal controls (Figure 1B); the decreases were principally associated with stage I/II PDAC (Figure 1C).

### Netrin-1 inhibits PDAC xenograft growth

**Figure 2:**
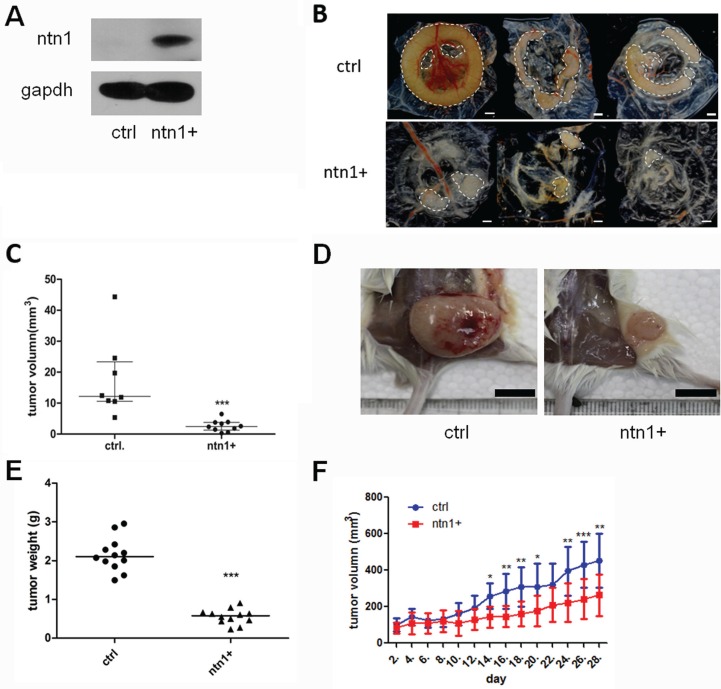
Netrin-1 inhibits the growth of MiaPaCa II xenograft tumors (**A**) Netrin-1 over-expression in MiaPaCa II cells was determined by western blotting with an anti-Netrin-1 antibody (sc-9292, Santa Cruz Biotechnology). Control (ctrl) cells were infected with a retrovirus without the netrin-1 CDS insertion, and GAPDH was used as an internal control. (**B**) Xenograft tumors of the control MiaPaCa II cells (ctrl) and netrin-1-over-expressing cells (ntn1+) in a chicken chorioallantoic membrane (CAM) model (bar, 1 mm). (**C**) Statistics for the size of the CAM xenograft tumors formed by the control cells (*N* = 8) and netrin-1-over-expressing cells (*N* = 10). The lines refer to the median size of each group (***P < 0.001). (**D**) Xenograft tumors formed by the control (ctrl) and netrin-1-over-expressing MiaPaCa II cells (ntn1+) in the SCID-beige mice (bar, 1 cm). (**E**) Statistics for the weights of the xenograft tumors formed by the control (*N* = 12) and netrin-1-over-expressing cells (*N* = 12). The lines refer to the median weight of each group (***P < 0.001). (**F**) The growth curve of the mouse xenograft tumors formed by the netrin-1-over-expressing cells (*N* = 9) showed significant growth arrest compared with that of the control xenografts (*N* = 8). Volume of xenograft tumors on both chicken CAMs and mice was calculated using the formula v = 0.5 ab^2^ (a: long diameter, b: short diameter). (error bars represent the standard deviation of the measurements, *P < 0.05, **P < 0.01, ***P < 0.001).

To investigate the function of netrin-1 in the tumor-forming process of PDAC cells, netrin-1 was over-expressed in the PDAC cell line MiaPaCa II (Figure 2A). The netrin-1-over-expressing and vehicle-transfected MiaPaCa II cells were xenografted onto the chorioallantoic membrane (CAM) of chicken embryos and into SCID-beige mice. The tumor size was measured on day 7 in the CAM model. The tumors formed by the netrin-1-over-expressing cells were significantly smaller than those from the control cells (Figure 2B–2C). The tumors from the SCID-beige mice were collected and weighed after 30 days of xenograft growth. Similarly, netrin-1 over-expression led to a significantly decreased tumorigenicity of the MiaPaCa II cells in the mouse model (Figure 2D–2E). In addition, the tumor growth curves were delineated by measuring the tumor volumes of the xenografts in the nude mice (Figure 2F), and a successive 28-day examination showed a sustained lag in the tumor growth of the netrin-1-over-expressing cells. Together, these data indicate that netrin-1 inhibits the xenograft growth of PDAC tumors.

### Netrin-1 does not affect angiogenesis in PDAC xenografts

Netrin-1 also functions as an angiogenic cue [13]. To clarify whether netrin-1 disrupts angiogenesis to inhibit PDAC growth, we used immunohistochemical staining for the endothelial marker CD31 to investigate angiogenesis in the control and netrin-1-over-expressing MiaPaCa II xenografts (Supplementary Figure S1A). A quantitative analysis of the CD31 staining revealed no significant difference in vascular density between netrin-1-over-expressing and control xenografts, indicating that netrin-1 did not inhibit PDAC xenograft growth by modulating angiogenesis (Supplementary Figure S1B).

### Netrin-1 decreases the adhesion of PDAC cells and arrests the 3D growth of the cells in Matrigel

**Figure 3:**
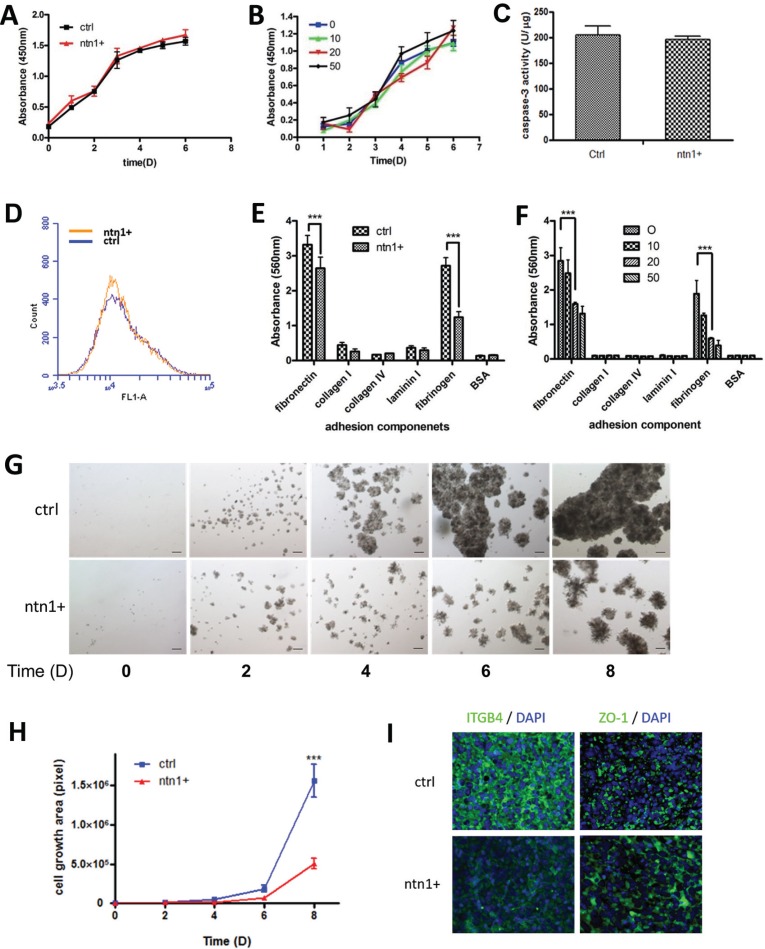
Netrin-1 suppresses ECM adhesion and the 3D growth of MiaPaCa II cells but does not affect proliferation and apoptosis in 2D cultures (**A**–**B**) Growth curve analysis of 2D-cultured netrin-1-over-expressing (ntn1+) and control (ctrl) MiaPaCa II cells (A) or MiaPaCa II cells treated with the indicated concentrations (ng/ml) of recombinant netrin-1 (B). No significant differences in cell proliferation were observed following either netrin-1 over-expression or recombinant netrin-1 protein treatment (*P* > 0.05). (**C**–**D**) Analysis of caspase-3 activity (C) and TUNEL assay (D) of the netrin-1-over-expressing (ntn1+) and control MiaPaCa II cells (ctrl). No significant differences in apoptosis were observed in either assay. (**E**–**F**) Quantification of the adhesion of the control (ctrl) and netrin-1-over-expressing (ntn1+) MiaPaCa II cells (E) or MiaPaCa II cells pretreated with the indicated concentrations (ng/ml) of recombinant netrin-1 for 24 hr (F) to the designated ECM components (***P < 0.001). (**G**–**H**) Micrographs (G) and growth curves (H) of the control (ctrl) and the netrin-1-over-expressing (ntn1+) MiaPaCa II cells cultured in 3D in Matrigel for 8 days (magnification, 200×; bar, 50 μm) (***P < 0.001). (**I**) Immunohistochemical staining of integrin β4 (ITGB4) and ZO-1 in the mouse xenograft tumor sections from the control (ctrl) and netrin-1-over-expressing (ntn1+) MiaPaCa II cells (magnification, 200×).

To investigate the mechanism by which netrin-1 inhibits PDAC tumor growth, we examined the control and netrin-1-over-expressing MiaPaCa II cells for their potential differences in proliferation, apoptosis, and adhesion ability. A growth curve assay showed no significant difference in MiaPaCa II cell proliferation in 2D cultures with either exogenously expressed netrin-1 or the administration of 10, 20 and 50 ng/ml of recombinant netrin-1 protein (Figure 3A–3B). Netrin-1 affects caspase-3 activity in certain tumor cells and inhibits apoptosis [7]. However, we failed to detect a significant change in caspase-3 activity in the netrin-1-over-expressing MiaPaCa II cells (Figure 3C). TUNEL staining confirmed that apoptosis was not affected in the netrin-1-over-expressing MiaPaCa II cells (Figure 3D).

An adhesion assay showed that netrin-1 over-expression and recombinant netrin-1 protein treatment significantly decreased the adhesion of MiaPaCa II cells to the ECM components fibronectin and fibrinogen, which show the highest affinity to MiaPaCa II cells (Figure 3E–3F). Moreover, the netrin-1-over-expressing MiaPaCa II cells displayed significantly impaired 3D growth in Matrigel matrix compared with the control cells (Figure 3G–3H). Similar results were obtained using another PDAC cell line, AsPC-1 (Supplementary Figure S2A–S2H). Interestingly, fluorescent immunohistochemical staining revealed increased staining for the epithelial marker ZO-1 in the netrin-1-over-expressing xenografts as well as sharply decreased staining for integrin β4 (ITGB4), a component of integrin α6β4, which is the previously reported netrin-1 receptor in the pancreas and one of the main mediators of cell-matrix adhesion of epithelial cells (Figure 3I).

Together, these data implied that netrin-1 suppresses PDAC progression by interfering with cell-matrix adhesion, and the process may involve modulated integrin β4 expression.

### Netrin-1 inhibits PDAC growth by decreasing integrin β4 expression

**Figure 4:**
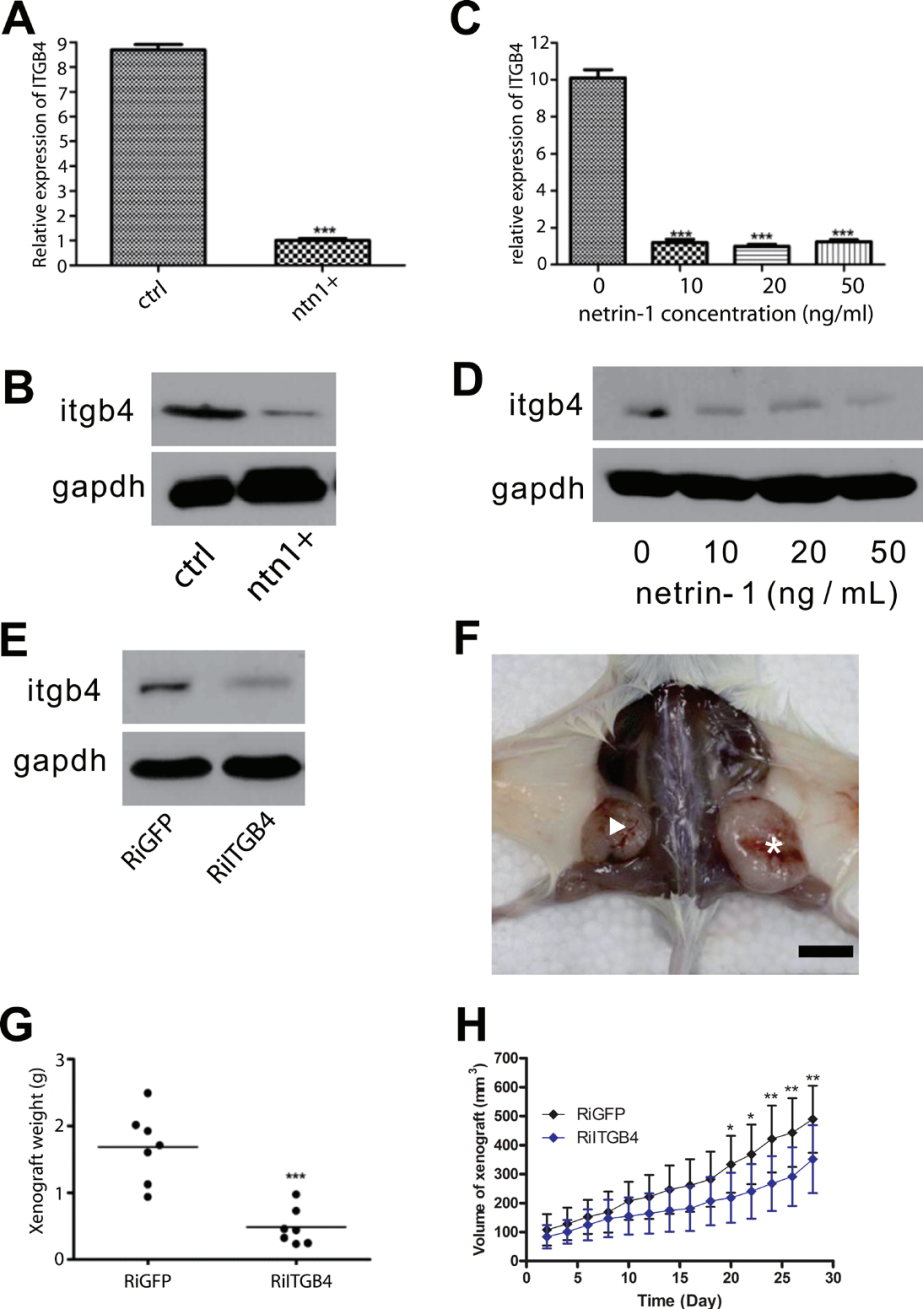
Netrin-1 inhibits PDAC growth by decreasing integrin β4 expression (**A**–**B**) Real-time RT-PCR (A) and western blotting (B) analyses of integrin β4 expression in control (ctrl) and netrin-1-over-expressing MiaPaCa II cells (ntn1+). GAPDH was used as the internal control for both analyses. (**C**–**D**) Real-time RT-PCR (C) and western blotting (D) analyses of integrin β4 expression in MiaPaCa II cells treated with the indicated concentrations of netrin-1 for 48 hr. GAPDH was used as the internal control for both analyses. (**E**) Western blotting analysis of integrin β4 expression in control GFP RNAi (RiGFP) and ITGB4 RNAi (RiITGB4) MiaPaCa II cells. GAPDH was used as the internal control. (**F**) Representative xenograft tumors formed by GFP RNAi (asterisk) and ITGB4 RNAi (arrowhead) MiaPaCa II cells in SCID-beige mice (bar, 1 cm). (**G**) Statistics for the weights of the xenograft tumors formed by the control RiGFP (*N* = 7) and RiITGB4 (*N* = 7) MiaPaCa II cells (***P < 0.001) in SCID-beige mice. (**H**) Growth curves of the RiGFP and RiITGB4 MiaPaCa II xenograft tumors in Balb/c NU/NU mice. The tumor volumes were measured every other day and calculated using the formula v = 0.5 ab^2^ (a: long diameter; b: short diameter); the total 28-day result is shown (error bars represent the standard deviation of the measurements, *P < 0.05, **P < 0.01).

We investigated whether netrin-1 could decrease integrin β4 expression in PDAC cells, as indicated by the immunohistochemical staining assay. Real-time RT-PCR and western blotting were performed to determine the integrin β4 expression levels in netrin-1-over-expressing (Figure 4A–4B) and recombinant netrin-1-treated MiaPaCa II cells (Figure 4C–4D). The results showed that netrin-1 dramatically reduced integrin β4 expression, as expected. Similar results were obtained using another PDAC cell line, AsPC-1 (Supplementary Figure S3A–S3C).

Integrin β4 is critical for epithelial integrity and has been reported to promote tumorigenesis in several different tumor types [18, 21, 22]. The role of integrin β4 in PDAC progression was examined via the RNA interference of integrin β4 expression in MiaPaCa II cells (Figure 4E) followed by a xenograft analysis of the control and integrin β4-knockdown cells in immunodeficient mice. The integrin β4-knockdown cells exhibited a significantly reduced tumor-forming ability (Figure 4F–4H), indicating that integrin β4 is actively involved in regulating PDAC progression and at least partially mediates the netrin-1-induced suppression of PDAC.

### Netrin-1 interacts with the UNC5b receptor to down-regulate integrin β4 expression

**Figure 5:**
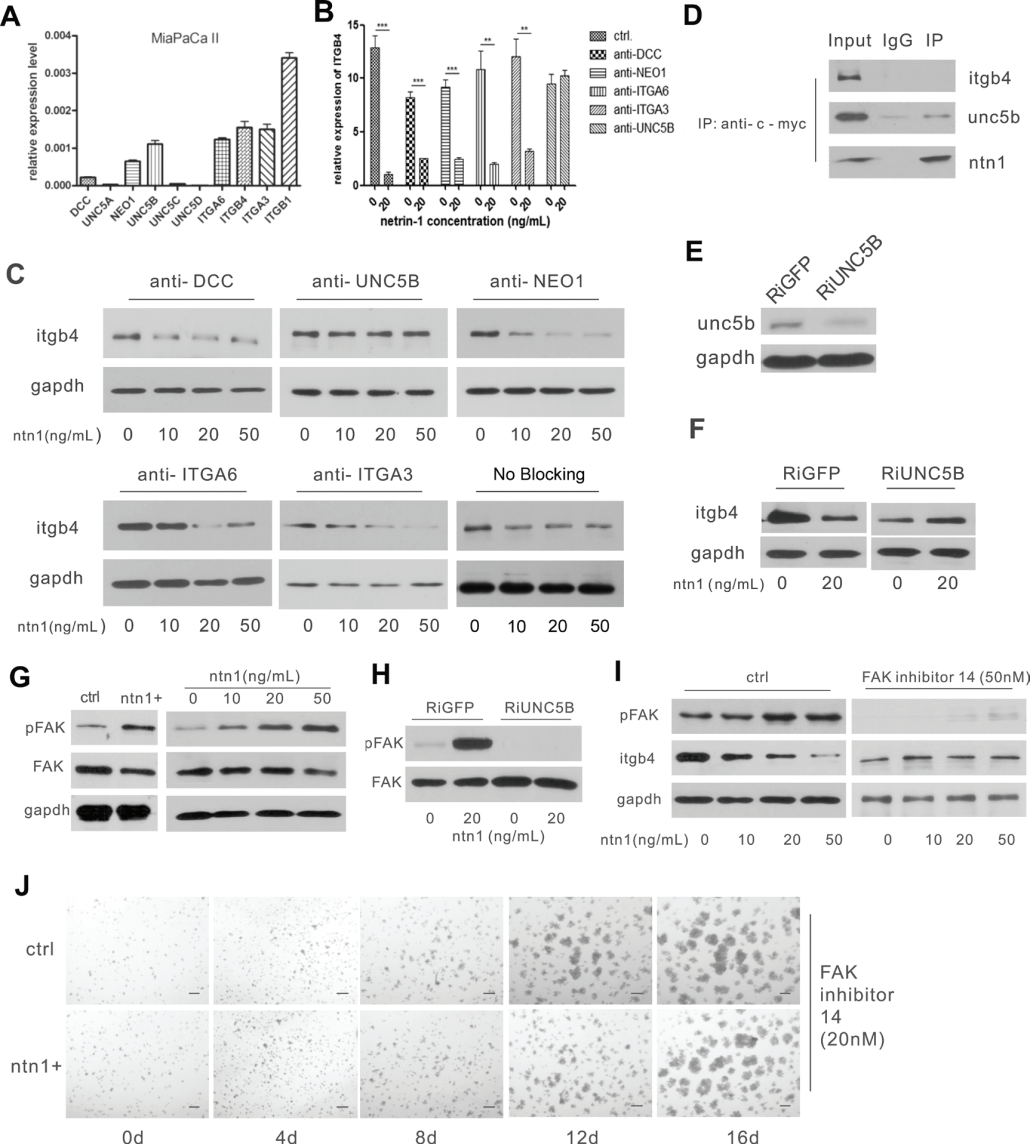
Netrin-1 down-regulates integrin β4 expression through the UNC5b receptor and the activation of FAK (**A**) Real-time PCR analysis for the expression of netrin-1 receptors in MiaPaCa II cells. GAPDH was used as an internal control. (**B**–**C**) The anti-UNC5b antibody selectively blocks the integrin β4-suppressing effect of netrin-1. The receptors on the MiaPaCa II cells were blocked by their respective antibodies before the cells were treated with the indicated concentrations netrin-1; integrin β4 expression in the cells was then detected by real-time RT-PCR (***P < 0.001, **P < 0.01) (B) and western blotting (C). GAPDH was used as an internal control. (**D**) Netrin-1 interacts with UNC5b but not integrin β4 in the myc-netrin-1-over-expressing MiaPaCa II cells. An anti-myc antibody to pull down the myc-tagged netrin protein in the netrin-1-over-expressing MiaPaCa II cells, followed by immunoblotting analysis of the integrin β4 (itgb4), UNC5b and netrin-1 (ntn1) levels in the precipitate. (**E**) Western blotting analysis of UNC5b expression in the UNC5B RNAi (RiUNC5B) and control GFP RNAi (RiGFP) MiaPaCa II cells. (**F**) Netrin-1 suppresses integrin β4 expression in the control GFP RNAi cells but not the UNC5B RNAi cells. Unc5b-knockdown and control MiaPaCa II cells were treated with recombinant netrin-1, and the integrin β4 expression was detected before and after netrin-1 treatment by western blotting. (**G**) Western blotting analysis of the FAK and phospho-FAK levels in the netrin-1-over-expressing MiaPaCa II cells, and the MiaPaCa II cells treated with the indicated concentrations of recombinant netrin-1 for 2 hr. The vector-transfected and the untreated MiaPaCa II cells were used as the respective negative controls, and GAPDH was used as an internal control. (**H**) UNC5b RNAi abrogates netrin-1-induced FAK phosphorylation. The FAK and phospho-FAK levels in the control and UNC5b RNAi MiaPaCa II cells were detected before and after netrin-1 stimulation by western blotting. (**I**) FAK inhibition abrogates the netrin-1-induced down-regulation of integrin β4. Western blot analysis of the phospho-FAK and integrin β4 levels in MiaPaCa II cells treated with the indicated concentrations of recombinant netrin-1 in the presence or absence of FAK inhibitor 14. (**J**) FAK inhibition abrogates the netrin-1-induced growth arrest of MiaPaCa II cells in Matrigel, as analyzed by the 3D growth of the control and netrin-1-over-expressing MiaPaCa II cells in Matrigel in the presence of 20 nM FAK inhibitor 14. Scale bars, 50 μm.

Integrin α6β4 has also been demonstrated as a receptor for netrin-1 in branching morphogenesis during the early development of the pancreas [14]. To determine whether netrin-1 suppresses integrin β4 expression by interacting with integrin α6β4 or other netrin-1 receptors, we first investigated the expression of known netrin-1 receptors in two PDAC cell lines, MiaPaCa II and AsPC-1 (Figure 5A, Supplementary Figure S4A). Four potential receptors—uncoordinated-5b (UNC5b), integrin α6β4, integrin α3β1 and neogenin—were expressed in both cell lines, whereas DCC and other uncoordinated (UNC) family members were expressed at substantially lower or undetectable levels in these cells.

Antibodies to the expressed receptors were then applied to block their interaction with netrin-1. Real-time RT-PCR (Figure 5B) and western blotting (Figure 5C) assays showed that the blocking antibodies to the UNC5b receptor selectively prohibited the integrin β4-suppressing effect of netrin-1 in MiaPaCa II cells. A co-immunoprecipitation assay with an anti-myc antibody, which recognized the myc-tagged netrin-1 that was over-expressed in the MiaPaCa II cells, precipitated UNC5b instead of integrin β4 (Figure 5D). Furthermore, knockdown of UNC5b expression in both MiaPaCa II and AsPC-1 cells by RNAi abrogated the observed down-regulation of integrin β4 following treatment with the recombinant netrin-1 protein (Figure 5E–5F; Supplementary Figure S4B–S4C). The selective interaction between the over-expressed myc-netrin-1 and UNC5b was also observed in the AsPC-1 cells (Supplementary Figure S4D).

Together, these data indicated that UNC5b was the receptor for netrin-1 and down-regulated integrin β4 in PDAC cells.

### Netrin-1/UNC5b activates focal adhesion kinase (FAK) to down-regulate integrin β4 expression

FAK is an important downstream effector of netrin-1. We detected increased FAK phosphorylation in both netrin-1-over-expressing and recombinant netrin-1-treated MiaPaCa II cells (Figure 5G). However, netrin-1 treatment of the UNC5b-knockdown MiaPaCa II cells failed to activate FAK, indicating that FAK activation occurs downstream of UNC5b (Figure 5H). The inhibition of FAK activity in MiaPaCa II cells with FAK inhibitor 14 abrogated the netrin-1-induced integrin β4 down-regulation and 3D growth arrest of the cells in Matrigel (Figure 5I–5J). The same inhibitor also blocked the netrin-1 mediated suppression of integrin β4 in AsPC-1 cells (Supplementary Figure S5). These results demonstrate that FAK is the key signal transduction molecule downstream of netrin-1/UNC5b that regulates the expression of integrin β4 and suppresses PDAC tumorigenesis.

### Netrin-1 enhances PP2A activity and suppresses the MEK/ERK/c-Jun pathway to down-regulate integrin β4 expression

**Figure 6:**
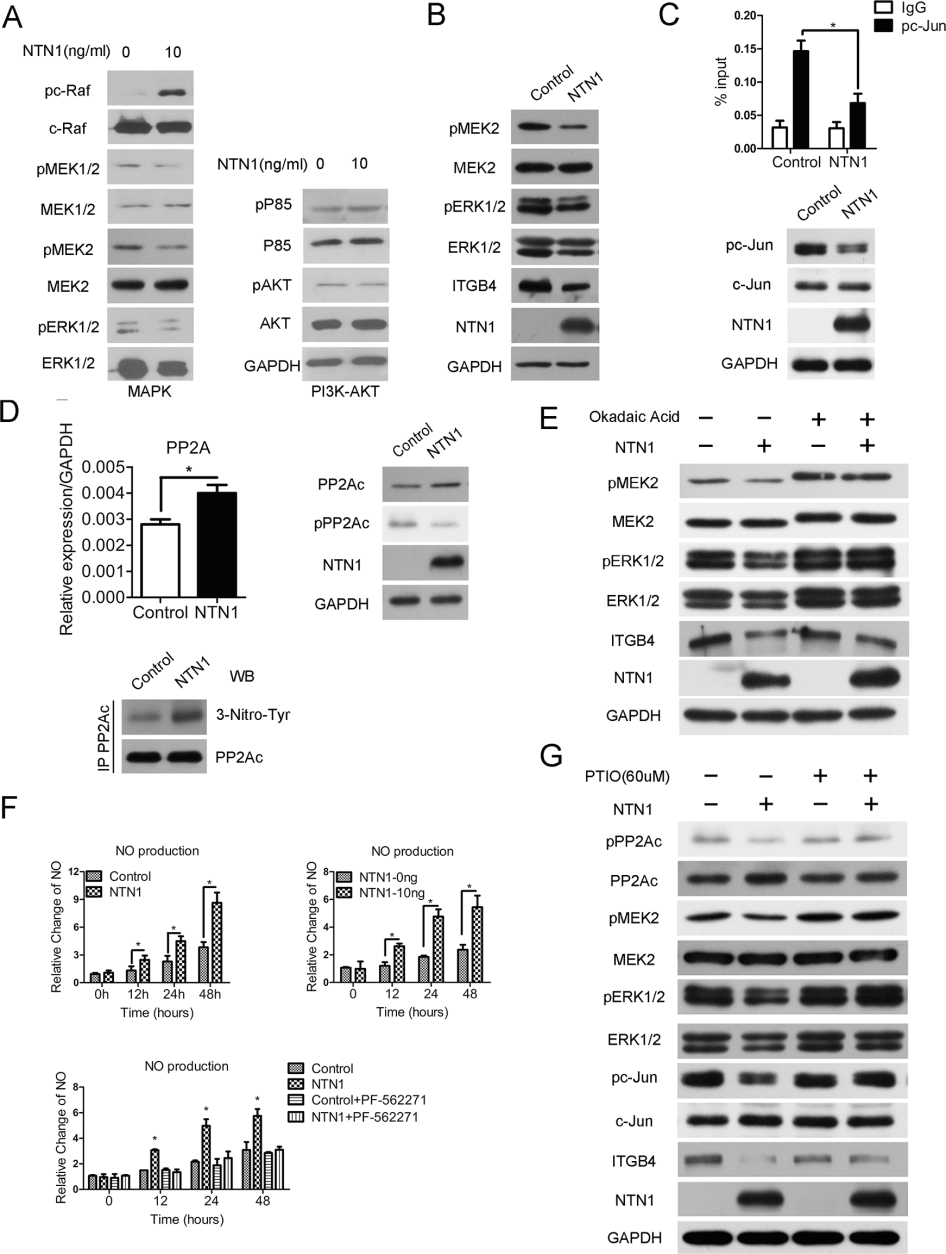
Netrin-1 induces nitric oxide (NO)-stimulated PP2A activation and suppresses the MAPK pathway to down-regulate integrin β4 expression (**A**) The western blotting analysis of the MAPK pathway (C-RAF/MEK/ERK) and PI3K-AKT pathway in the netrin-1-treated MiaPaCa II cells shows that MEK/ERK signals in the MAPK pathway were reduced and the PI3K-AKT pathway was unchanged. (**B**) Western blotting assay showing reduced MEK/ERK signals and decreased integrin β4 expression in the netrin-1-over-expressing MiaPaCa II cells. (**C**) A ChIP analysis (upper panel) and western blotting assay (below panel) of the phosphorylated c-Jun (pc-Jun) levels in the netrin-1-over-expressing MiaPaCa II cells showed reduced pc-Jun levels and decreased pc-Jun recruitment to the integrin β4 gene promoter upon netrin-1 over-expression. (*P < 0.05). (**D**) RT-PCR and western blotting assay showing increased PP2A expression and decreased phosphorylation and enhanced 3-nitrotyrosine modification of PP2A in the netrin-1-over-expressing MiaPaCa II cells. (*P < 0.05). (**E**) A western blotting assay of the netrin-1-over-expressing MiaPaCa II cells treated with the PP2A inhibitor okadaic acid showed that the repression of MEK/ERK signaling and integrin β4 expression depends on PP2A activation. (**F**) NO production was increased in the netrin-1-over-expressing and recombinant netrin-1-treated MiaPaCa II cells. FAK inhibitor PF-562271 blocks the netrin-1-induced NO production. (*P < 0.05). (**G**) Western blotting assay of the netrin-1-over-expressing MiaPaCa II cells treated with the NO scavenger PITO. The NO scavenger abolished the netrin-1-induced changes in PP2A expression and phosphorylation, the MEK/ERK signals, c-Jun phosphorylation and integrin β4 expression. GAPDH was used as an internal control for all western blotting assays.

To further delineate the netrin-1/UNC5b/FAK signaling pathway that suppresses integrin β4 expression, the activities of two pathways downstream of FAK—the MAPK (RAF/MEK/ERK) and PI3K-AKT pathways—were examined following netrin-1 treatment. The western blotting assay revealed that MAPK signaling, and not the PI3K-AKT pathway, was disrupted. Interestingly, although c-RAF phosphorylation is activated by netrin-1 treatment, the downstream kinase MEK was markedly suppressed, particularly the MEK2 isoform, followed by impaired ERK1/2 phosphorylation and a simultaneous decrease in ERK1/2 protein levels (Figure 6A). Similar results were obtained with the netrin-1-over-expressing cells (Figure 6B). Integrin β4 was previously shown to be up-regulated by JNK/AP-1, which is downstream of MEK/ERK signaling [23]. Both c-Jun phosphorylation and its recruitment to the ITGB4 gene promoter were decreased in the netrin-1-over-expressing cells, consistent with the down-regulation of integrin β4 (Figure 6C).

We next sought to understand the mechanisms leading to the suppression of MEK/ERK signaling. In addition to the canonical RAF/MEK/ERK cascade that promotes MEK/ERK phosphorylation and activation, MEK/ERK are primarily dephosphorylated and suppressed by the protein phosphatase 2A (PP2A). As the upstream c-RAF kinase is activated by netrin-1, we analyzed the dephosphorylation of the pathway and detected increased expression of PP2A at both the RNA and protein levels, as represented by its catalytic subunit (PP2Ac). The phosphorylation of PP2Ac, which is inversely correlated with PP2A activity, was obviously decreased, despite the enhanced PP2A expression, suggesting that the PP2A pathway was significantly activated (Figure 6D, upper panels). Inhibition of PP2A activity with okadaic acid blocked the observed MEK/ERK suppression and largely relieved the netrin-1-induced down-regulation of integrin β4 (Figure 6E). These data demonstrate that netrin-1 down-regulates integrin β4 by strengthening the PP2A-mediated inhibition of the MAPK (MEK/ERK) pathway.

### Netrin-1/UNC5b/FAK stimulates nitric oxide (NO) production to promote PP2A activity

Finally, we investigated the signals transduced from netrin-1/UNC5b/FAK that activate PP2A. NO is a signaling molecule that is downstream of netrin-1 [24] and was reported to augment PP2A phosphatase activity via nitrification of the enzyme at Tyr284 [25, 26]. A consistent increase in NO production was detected in the netrin-1-over-expressing and recombinant netrin-1-treated MiaPaCa II cells. The FAK inhibitor PF-562271 abrogated the netrin-1-induced NO production (Figure 6F). We also detected an increase in the 3-nitrotyrosine modification of PP2A in the netrin-1-over-expressing cells (Figure 6D, lower panel). Moreover, pretreatment of the cells with the NO scavenger PITO (2-phenyl-4,4,5,5- tetramethylimidazoline-1-oxyl-3-oxide) abolished the netrin-1-over-expression-induced PP2A activation and the MEK/ERK/c-Jun inhibition and largely restored the integrin β4 expression in the netrin-1-over-expressing cells (Figure 6G). Similar results were observed in the cells treated with L-NAME (N-nitro-L-arginine methyl ester), a nitric oxide synthase inhibitor (Supplementary Figure S6). These results indicate that NO production is required for netrin-1 to promote PP2A activity, restrain MEK/ERK signaling and inhibit integrin β4 expression.

## DISCUSSION

In the present study, we found that netrin-1 expression was decreased in stage I/II PDAC samples, and demonstrated that netrin-1 suppressed the tumor-forming ability of PDAC cells. We showed that netrin-1 inhibited PDAC tumor growth by interfering with integrin β4 expression, which depends on the activation of UNC5b/FAK signaling and requires NO-mediated PP2A activation and the subsequent suppression of the MAPK pathway.

Using immunohistochemical staining, we characterized the expression pattern of netrin-1 during PDAC progression and observed netrin-1 staining in the exocrine acini of the normal pancreatic tissue. This observation is in agreement with Yebra's work using mixed ducts/acini tissue from adult pancreas [14]. Interestingly, we witnessed a clear decrease in netrin-1 expression in stage I/II PDAC samples compared with that in the normal pancreatic tissue. Decreased netrin-1 expression has also been described in prostate and brain cancers [27, 28]. Acinar cells are now considered one of the main origins for the metaplastic ductal epithelium of PDAC [29, 30]. The concomitant loss of acinar netrin-1 expression and the metaplasia of acinar cells in the early-stage PDAC samples support future investigations into a role of netrin-1 in PDAC initiation. We observed obviously elevated netrin-1 expression in the ducts of advanced-stage PDAC, in agreement with previous reports [15, 16].

We showed that netrin-1 inhibited 3D growth and the tumorigenesis of xenografted PDAC cells, which suggested a role for netrin-1 in suppressing integrin β4 expression. Accumulating evidence has suggested that integrin β4 over-expression promotes tumor development and progression [31–33]. Breast cancer cells that over-express the signaling-defective integrin β4-1355T mutant mimic the netrin-1-over-expressing cells used here in the arrested tumor growth *in vivo* and the unaffected cell proliferation in normal cultures *in vitro* [21]. Integrin β4 expression is obviously increased and redistributed in PDAC tumors and has been identified as one of the earliest markers of PDAC [20]. A tumor-suppressive role of netrin-1 was also observed in several other types of tumor cells [34–36]. Previous studies reported that netrin-1 promotes the adhesion, survival and invasion of PDAC cells and that netrin-1 is associated with a worse prognosis of PDAC patients [15, 16]. Our work concerns the *in vivo* growth of PDAC cells rather than the invasion and metastasis of advanced PDAC. Moreover, the differences in the PDAC cell types and the netrin-1 receptors or co-receptors that are expressed might account for the contrasting findings regarding the role of netrin-1 in PDAC progression. As noted above, opposite roles of netrin-1 have also been reported in angiogenesis [11–13]. Together, these results suggest that netrin-1 influences tumorigenesis in a context-dependent manner. Whether and how the netrin-1-induced down-regulation of integrin β4 could be applied to other epithelial malignancies and netrin-1-related developmental events are intriguing questions that require further investigation.

A previous study showed that integrin α6β4 functions as a receptor for netrin-1 during pancreas morphogenesis. In this study, we observed that integrin β4 is the target, instead of the receptor, of netrin-1 and demonstrated that canonical UNC5b is the receptor. We could not exclude the possibility that integrin β4 functions as a netrin-1 receptor in the PDAC cells during other signaling events. FAK is one of the main downstream effectors of netrin-1 through the receptors DCC and neogenin [37–41]. Intimate crosstalk between the UNC5 receptor and FAK was observed. Both FAK and Src, a common partner of FAK, are necessary for the netrin-induced phosphorylation of UNC5 [39]. A recent study showed that UNC5b modulates the activity of Src [42]. In the present study, we detected significant FAK phosphorylation that was abolished by UNC5b knockdown in netrin-1-stimulated PDAC cells, suggesting that UNC5b also regulates FAK activity.

Abnormal activation of the RAF/MEK/ERK cascade is tightly associated with tumorigenesis including PDAC. Activation of the MAPK signaling pathway was important for the chemoattractive effect of netrin-1, whereas the repulsive signaling from the Eph family receptors repressed MAPK signaling [43, 44]. Interestingly, in this study, we found that netrin-1, which activates c-RAF upstream of the MEK/ERK signaling, also stimulates NO production and remarkably increases PP2A activity, eventually suppressing MEK/ERK signaling. The repressive effect of netrin-1 on the MAPK pathway was mediated by the repulsive UNC5b receptor, and our preliminary data showed that another netrin-1 receptor, neogenin, was required for the observed c-RAF activation in the present study (Supplementary Figure S7). Netrin-1-induced inhibition of ERK signaling was observed previously during lung branching morphogenesis, which likely occurred through the UNC5b receptor as well [45], implying a general impact of this regulatory pathway. Thus, the bidirectional impacts of netrin-1 on the MEK/ERK pathway activity may provide a mechanistic basis for the finely balanced effects of netrin-1 in PDAC tumorigenesis.

**Figure 7:**
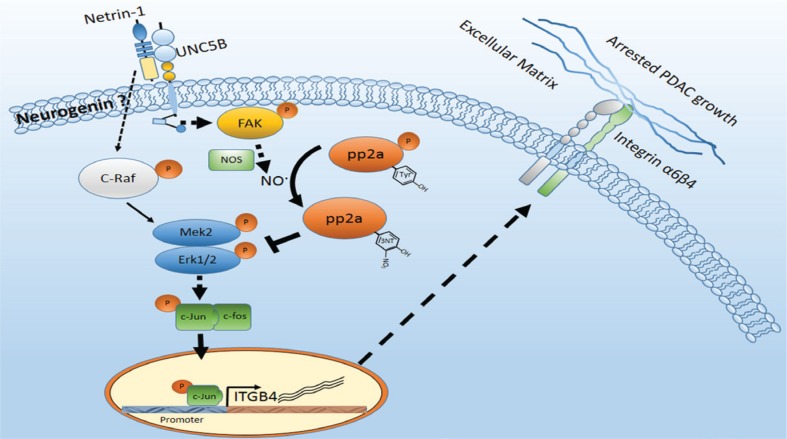
Schematic model of the netrin-1-induced growth inhibition of PDAC Netrin-1 is expressed in the exocrine acini of normal pancreatic tissue, and this expression is impaired in early-stage PDAC. Netrin-1 inhibits PDAC tumorigenesis by interacting with the UNC5b receptor and stimulating FAK phosphorylation. Activated FAK induces the production of nitric oxide (NO) and promotes the expression, 3-nitrotyrosine modification and dephosphorylation of the protein phosphatase PP2A, leading to significantly enhanced PP2A activity. PP2A suppresses MEK/ERK/c-Jun signaling and down-regulates integrin β4 expression, facilitating the *in vivo* growth arrest of PDAC cells. Interestingly, in this study, that netrin-1 also mildly induced c-Raf phosphorylation, which could promote MEK/ERK signaling. Together, our data suggest that netrin-1 protects against PDAC and, at the same time, imply that netrin-1 intricately regulates PDAC progression in different cells and/or distinct stages of PDAC tumorigenesis.

Taken together, our data indicate a suppressive role for netrin-1 in the *in vivo* growth of PDAC cells, as shown in the working mechanism (Figure 7). Considering the pro-tumorigenic effect of netrin-1 in advanced stages, the therapeutic potential of netrin-1 in PDAC still requires careful investigation and evaluation.

## MATERIALS AND METHODS

### Cell lines and animal strains

MiaPaCa II, AsPC-1, and 293T cells were preserved by our laboratory and identified by STR profiling. The chicken embryos for the xenograft growth assay were specific pathogen-free (SPF) eggs (Merial Vital Co., LTD., male and female), and four-week-old SCID-beige mice (Vital River Co., LTD., 15–20 g, males) were used for the mouse xenograft growth assay. Balb/c NU\NU mice (Vital River Co., LTD., four-week, 15~20 g, male) were used for the xenografts growth curve assay. All animal protocols were approved by the Animal Care and Use Committee at the Institute of Basic Medical Sciences, Chinese Academy of Medical Sciences and Peking Union Medical College (No. 2014-5-12-973).

### Microarray of human PDAC and normal pancreas samples

The PATMA012 microarrays of the pancreatic cancer and normal pancreas tissue samples were obtained from Creative Bioarray (NY, USA). This array contains 80 samples: 30 stage-I, 24 stage-II, 3 stage-III, and 4 stage-IV ductal adenocarcinoma samples and 10 normal pancreas tissue samples. The remaining samples are from patients with other types of pancreatic malignancies. Each core was from a separate case, and all samples were fixed in formalin. The sample cores were sectioned into 5-μm-thick slices with a diameter of 1.5 mm; these sample cores were coated with paraffin.

### Cell culture and retrovirus generation

MiaPaCa II, AsPC-1 and 293T cells were cultured in Dulbecco's Modified Eagle Medium (DMEM), high glucose (11995–073, Gibco^®^, Life Technologies) with 10% fetal bovine serum (FBS, 10100147, Gibco^®^, Life Technologies). The cells were grown in monolayers in a humidified atmosphere of 5% CO_2_ at 37°C, and the medium was changed every other day until the cells reached confluence. The subculturing methods for these cells followed the suggested methods with trypsin-EDTA (25300–054, Gibco^®^, Life Technologies). The cells were cryopreserved in liquid nitrogen in DMEM with 10% DMSO (D12345, Molecular Probes^®^, Life Technologies) and 20% FBS.

Fresh DMEM medium (with 10% FBS) containing the indicated concentrations of recombinant netrin-1 protein was applied to the cells daily in the indicated experiments. FAK inhibitor 14 was added to serum-free DMEM medium at a concentration of 50 mM for a 2-hr treatment. FAK inhibitor PF-562271 was used to treat Miapaca II cells at a concentration of 1μM for 48 hours. Carboxy-PTIO and NOS inhibitor L-NAME were used at a concentration of 60 μM and 30 mM respectively for 48 hours. Okadaic acid was used at a concentration of 1 μM for 6 hours before cell collection.

Netrin-1 over-expression retroviral vectors were constructed by inserting the coding sequence of *netrin-1* (NM_004822.2) with *c-myc* sequence tagged at the C-terminal into the pMSCV vector (Clontech Inc.). The control retroviral vector was the empty pMSCV vector. The netrin-1 expression and control vectors were then transfected into 293T cells with the viral coat vectors pV and pMD. The virus-containing suspension was harvested 48 hr later and used to infect PDAC cells in FBS-free DMEM medium for six hours. The RNAi retroviral vector for integrin β4 and UNC5b was constructed by inserting ITGB4 (NM_001005731.1) and UNC5B (NM_001244889.1) interfering sequence (Supplementary Table S1) into the pSIREN-Retro Q vector (631526, Clontech Inc.). The RNAi sequence targeting GFP was used to construct the control vector. The retroviruses were then assembled, harvested and infected as described above.

### Xenograft models

The chicken embryos were grown in humidified atmosphere at 37.5°C and 60% humidity. A total of 5 × 10^6^ MiaPaCa II cells were xenografted onto the CAM of day 10 embryos. The xenografts were harvested from the CAM of day 17 embryos. Four-week-old SCID-beige mice were used for the mouse xenograft growth assay. A total of 5 × 10^6^ MiaPaCa II cells were subcutaneously injected into the back of each mouse. The tumors were harvested after four weeks of culture and weighed immediately after resection. Balb/c NU/NU mice were used for the xenograft growth curve assay. A total of 5 × 10^6^ MiaPaCa II cells was subcutaneously injected into the back of each mouse, and the xenograft diameters were measured using a slide caliper every other day until day 28. The xenograft tumor volume was calculated using the following formula: v = 0.5 ab^2^ (a = the long diameter of the tumor, b = the short diameter of the tumor, and v = volume) [46, 47].

### Real-time RT-PCR

Total RNA was extracted using Trizol reagent (Invitrogen) according to the manufacturer's protocol. Two micrograms of total RNA was used for the first-strand synthesis with cDNA M-MuLV Reverse Transcriptase (New England Biolabs) using random primers. The Quantitect SYBR Green RT-PCR Kit (Qiagen) was employed for the amplification reactions using the StepOnePlus^®^ protocol described by the manufacturer with the Applied Biosystems^®^ Real-time PCR Detection System. The fluorescence curves were analyzed using StepOne Software (Version 2.1). The primers for real-time RT-PCR are listed in Supplementary Table S2.

### Immunoprecipitation, receptor block and western blot

The cells were lysed with an IP buffer (20 mM Tris pH 7.5, 150 mM NaCl, and 1% Triton X-100) in the presence of a proteinase inhibitor cocktail (P8340, Sigma-Aldrich, Inc.) and phenylmethylsulfonyl fluoride (PMSF, 1 mM). The lysates were clarified by centrifugation at 12,000 rpm for 30 min, and the protein concentration was measured using the BCA protein assay (Pierce, Thermo Fisher Inc.). The proteins (500 μg) were pre-cleared with Protein G-agarose beads (15920-010, Novex^®^, Life Technologies) and then incubated with the indicated antibodies or mouse IgG overnight at 4°C. The immune complexes were captured with Protein G-agarose beads for 3 hr at 4°C. The protein was eluted from the beads by boiling in protein loading buffer (P0015, Beyotime) before use in the western blotting assays.

The netrin-1 receptor was blocked using the method described by Yebra and colleagues [14]. Briefly, antibodies to known netrin-1 receptors were used to pretreat the cells at a concentration of 1 μg/ml in a 24-well plate for 2 hr before the indicated concentrations of netrin-1 were added to the medium. The cells were collected 24 hr later and subjected to western blotting. The cells were successively lysed and dissolved in radioimmunoprecipitation assay (RIPA) buffer (25 mM Tris-HCl, pH 7.6, 150 mM NaCl, 1% NP-40, 1% sodium deoxycholate, and 0.1% SDS) containing a proteinase inhibitor cocktail, PMSF (1 mM) and a phosphatase inhibitor cocktail (04906845001, Roche). After complete homogenization on ice in a rotator, the samples were sonicated and centrifuged at 4°C. The supernatants were transferred into fresh tubes, and the protein concentrations were determined using the BCA method. Equal amounts of protein (20 μg/lane) were separated by SDS-PAGE and transferred onto polyvinylidene difluoride membranes (Millipore). After blocking, the filters were incubated with the primary antibodies for western blotting. After being washed and incubated with the appropriate horseradish peroxidase-conjugated secondary antibody (Santa Cruz Biotechnology), the immune complexes were visualized with a chemiluminescence reagent.

### Immuno-histochemical staining

The xenografts were fixed in 4% paraformaldehyde phosphate-buffered saline and coated in paraffin. The samples were sectioned into five-μm-thick slices, and the sections were placed onto positively charged glass slides (ZLI-9506; Zhongshan Goldenbridge Biotechnology). For immuno-histochemical staining, the slides were deparaffinized, and endogenous peroxidase activity was quenched with 3% (v/v) hydrogen peroxide in 10% PBS for 10 min. Nonspecific binding sites were blocked with 10% bovine serum in PBS at room temperature for 1 hr. The slides were incubated at 4°C overnight with diluted primary antibodies and then with a biotinylated secondary antibody (Vector Laboratories) at 37°C for 30 min before subsequent incubation with a horseradish peroxidase-labeled streptavidin solution for 20 min at 37°C. The slides were stained with diaminobenzidine and counterstained with hematoxylin. In the fluorescent immuno-histochemical staining, fluorescent secondary antibodies (Alexa Fluor, Invitrogen) were applied. The protein content in the xenografts was analyzed by calculating the integration optical density value of positive staining within the media using Image Pro Plus software (Media Cybernetics).

### Cell cycle, adhesion and apoptosis assays

Growth curve assays were performed with the cell counting kit (CCK-8, Beyotime) daily for 7 days. The number of cells can be determined by the absorbance at 450 nm measured using a microplate reader (Synergy 4, Biotek). Caspase-3 activity was measured with a caspase-3 activity assay kit (C1115, Beyotime) following the user's manual; the absorbance at 405 nm was measured. The TUNEL assay was performed by applying a cell death detection kit (fluorescein, 11684795910, Roche), and the stained cells were counted and analyzed with a flow cytometry assay (Accuri C6, BD). The cell adhesion ability was measured by applying a Cytoselect^™^ cell adhesion assay kit (CBA-070, Cell Biolabs). The experimental process followed the kit manual; the absorbance at 560 nm was measured.

### Matrigel 3D tumorigenesis assay

In the Matrigel assay, 150 μl of an ice-cold Matrigel solution (1/2 Matrigel [356231, BD Biosciences] and 1/2 DMEM medium) was transferred into one well of a 24-well plate (Corning Co., Ltd.). The Matrigel was allowed to polymerize in a 37°C cell culture incubator for 30 min. Approximately 2,000 cells suspended in DMEM medium were added on top of the Matrigel in each well and cultured for the indicated times. The indicated concentrations of netrin-1 or drugs were added to the culture medium on top of the Matrigel. The tumorigenicity of the PDAC cells in Matrigel was observed and imaged using an inverted microscope (TE2000, Nikon).

### Nitric oxide examination

The production of nitric oxide was calculated by detecting the stable NO metabolite using the Griess reaction [48]. Fifty microliters of the culture supernatants was mixed with 50 μl of Griess reagent A (1% sulfanilamide and 6% H_3_PO_4_ (85%)) and incubated for 10 min at 37°C. Then, 50 μl of Griess reagent B (0.1% naphthyl ethylenediamine dihydrochloride) was added and incubated for 10 min at 37°C. The nitrite concentrations were determined by measuring the absorbance at 540 nm in an enzyme-linked immunosorbent assay reader.

### Drugs, recombinant protein and antibodies

The recombinant netrin-1 protein (#6419-N1-025/CF) used to treat the PDAC cells and the AsPC1 cells was purchased from R & D Systems. FAK inhibitor 14 (#3414) was purchased from TOCRIS Bioscience, and FAK inhibitor PF-562271 (S2890) was from Selleck Chemicals. The NOS inhibitor L-NAME (S0006), PP2A inhibitor okadaic acid (S1786) and NO scavenger Carboxy-PTIO (S1546) were from the Beyotime Institute of Biotechnology.

Anti-c-myc (sc-40, Santa Cruz Biotechnology) and anti-PP2A (ab32141, Abcam) antibodies were used for immunoprecipitation. Monoclonal antibodies against DCC (OP45, Calbiochem), Neogenin (sc-6537, Santa Cruz Biotechnology), UNC5b (unc5 h2, sc-28047, Santa Cruz Biotechnology), integrin α6 (sc-19622, Santa Cruz Biotechnology) and integrin α3 (CP11 L, Calbiochem) were used for the blocking assay to identify the netrin-1 receptors. Anti-netrin-1 (sc-9292, Santa Cruz Biotechnology) and anti-UNC5b (WH0219699M1, Sigma) antibodies were used for western blotting. The anti-integrin β4 (#4707), anti-GAPDH (#5174), anti-FAK (#3285), anti-phospho-FAK (#3283), anti-MEK1/2 (#9122), anti-phospho-MEK1/2 (S217/221, #9121), anti-ERK1/2 (#4695), anti-phospho-ERK1/2 (T202/Y204, #4370), anti-c-Jun (#9165), anti-phospho-c-Jun (S73, #3270), anti-phospho-P55 (Y199, #4228), anti-P55 (#11889), anti-phospho-Akt (T308, #13038), anti-Akt (#9272) and anti-Nitro-Tyrosine (#9691) antibodies used in the other western blot assays were purchased from Cell Signaling Technology. The anti-MEK2 (ab32517), anti-phospho-MEK2 (T394, ab131095), anti-PP2A alpha+beta (ab32141) and anti-phospho-PP2A-alpha (Y307, ab32104) antibodies were purchased from Abcam. The primary antibodies used for immuno-histochemical staining were Netrin-1 (ab122903, Abcam), CD31 (ab28364, Abcam), integrin β4 (ab110167, Abcam), and ZO-1 (ab59720, Abcam).

### Statistics

The statistical analyses were performed using SPSS version 13.0 (SPSS, Inc., Chicago, IL). The data are presented as the mean ± standard error of the mean (SEM) unless indicated otherwise. The paired data were analyzed using Student's *t*-test. Differences between groups were determined with one-way or two-way analysis of variance (ANOVA) with repeated measures, followed by the Bonferroni post hoc test. A probability value of less than 0.05 was considered significant.

## SUPPLEMENTARY MATERIALS FIGURES AND TABLES


