# Supplementary material for: Netrin-1 suppresses the MEK/ERK pathway and ITGB4 in pancreatic cancer
Source: Oncotarget. 2016 Mar 25;7(17):24719–33. doi: 10.18632/oncotarget.8348 (PMC5029736; doi:10.18632/oncotarget.8348)
Supplement: Supplementary file 1 [file oncotarget-07-24719-s001.pdf]

Netrin-1 suppresses the MEK/ERK pathway and ITGB4 in pancreatic cancer

Supplementary Materials

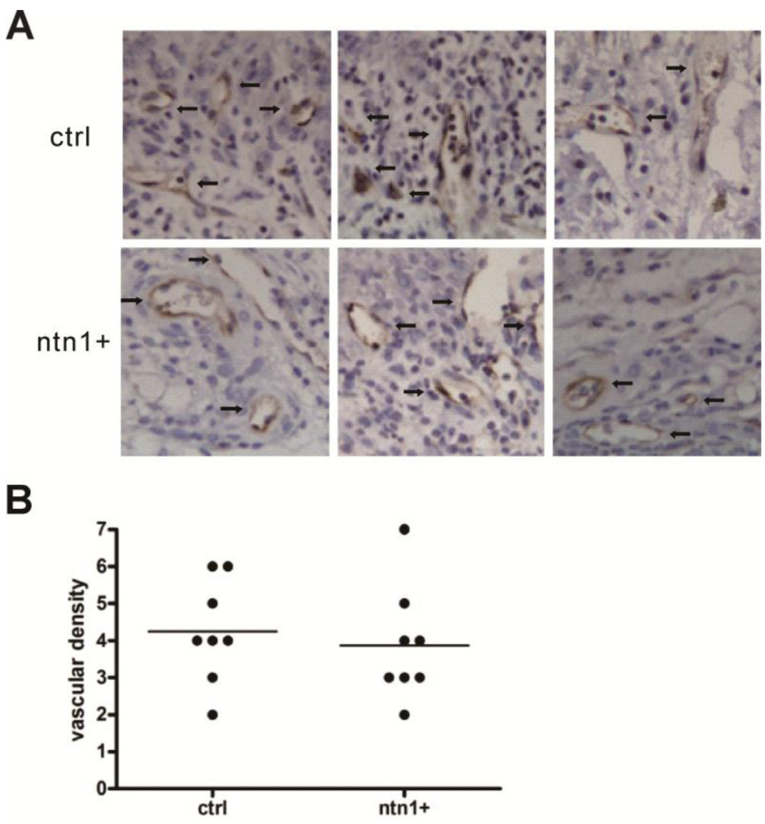

Supplementary Figure S1: Netrin-1 does not affect angiogenesis in MiaPaCa II xenograft tumors. (**A**) Vessel density in the control (ctrl) and netrin-1-over-expressing (ntn1+) MiaPaCa II xenograft tumors was determined by immunohistochemical staining with an anti-CD31 antibody (graphs are shown in 200×). Arrows indicate the sites of positive staining. (**B**) The vessel number within each microscopic field was counted and averaged; the statistical analyses showed no obvious differences in the vascular density between the netrin-1-over-expressing and control MiaPaCa II xenograft tumors.

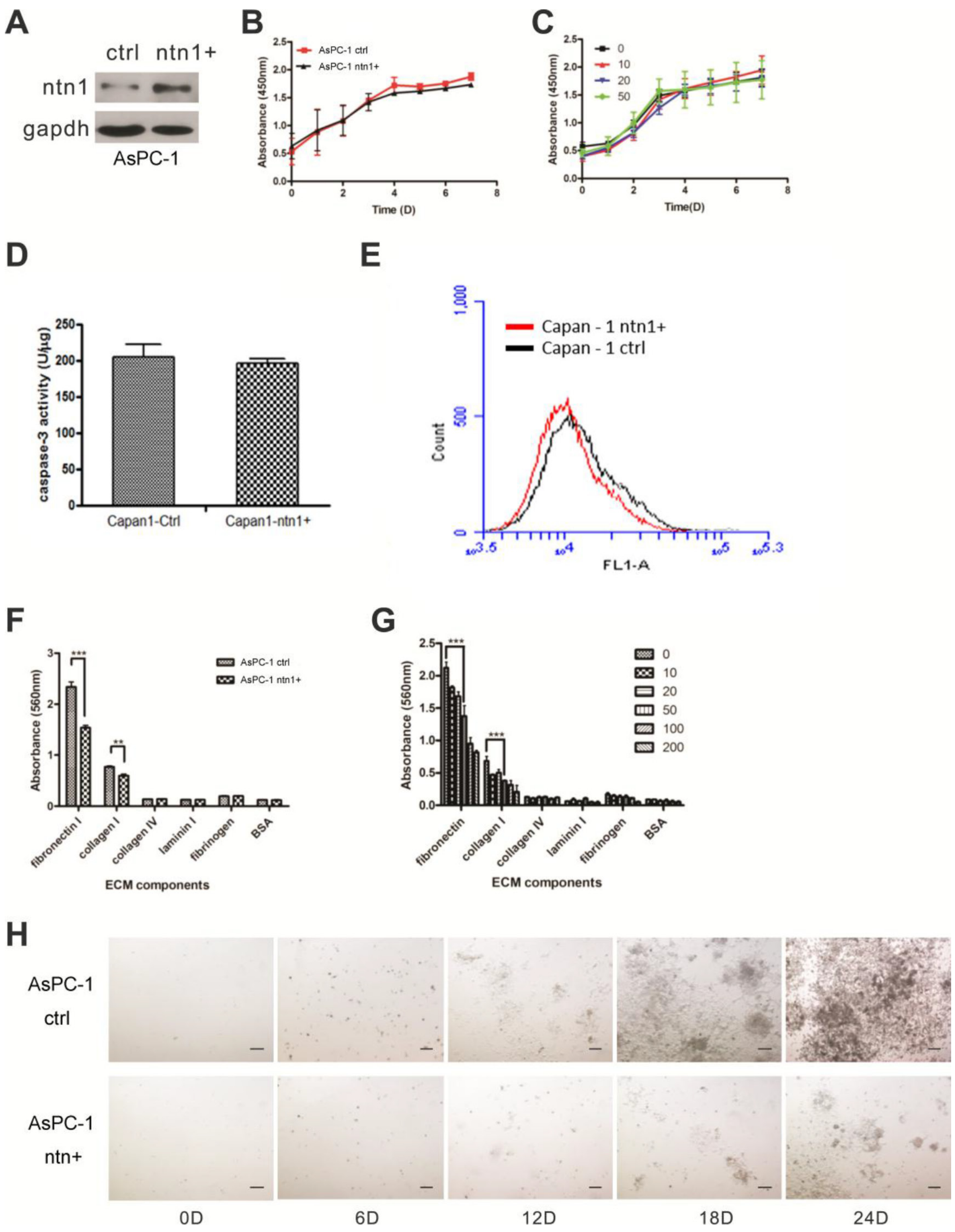

Supplementary Figure S2: Netrin-1 suppresses extracellular matrix (ECM) adhesion and the three-dimensional (3D) growth of AsPC-1 cells but did not affect proliferation and apoptosis in two-dimensional (2D) cultures. (**A**) Netrin-1 over-expression in AsPC-1 cells (ntn1+) was demonstrated by western blotting. The control cells (ctrl) were infected with retrovirus without netrin-1 CDS insertion, and GAPDH was used as an endogenous control. (**B**–**C**) Growth curve analysis of 2D-cultured netrin-1-over-expressing (AsPC-1 ntn1+) and the control (AsPC-1 ctrl) AsPC-I cells (B) or AsPC-1 cells treated with recombinant netrin-1 of the indicated concentration (ng/ml) (C). No significant difference in the cell growth curve was detected upon netrin-1 over-expression or recombinant netrin-1 treatment ($P > 0.05$). (**D**–**E**) Caspase-3 activity analysis (D) and TUNEL assay (E) of netrin-1-over-expressing (AsPC-1 ntn1+) and control AsPC-1 cells (AsPC-1 ctrl). No significant difference in apoptosis was observed between the netrin-1-over-expressing and control AsPC-1 cell lines. (**F**–**G**) Quantification of the adhesion of (F) control (AsPC-1 ctrl) and netrin-1-over-expressing (AsPC-1 ntn1+) AsPC-1 cells, or (G) AsPC-1 cells pretreated with recombinant netrin-1 at the indicated concentrations (ng/ml) for 24 hr, to the designated ECM components. (**H**) Micrographs of the control (AsPC-1 ctrl) and netrin-1-over-expressing (AsPC-1 ntn1+) AsPC-1 cells cultured in 3D in Matrigel for 24 successive days. Netrin-1 decreased the 3D growth of AsPC-1 cells in Matrigel. Scale bars, 50 μm.

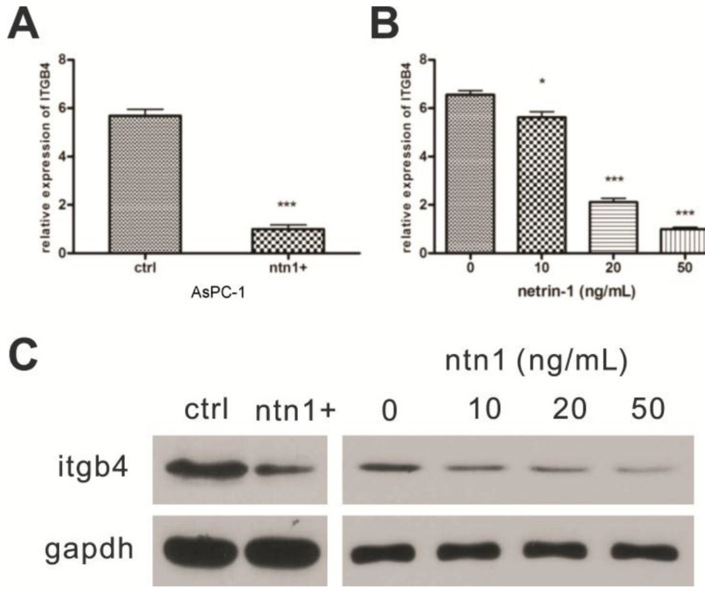

Supplementary Figure S3: Netrin-1 inhibits integrin β4 expression in AsPC-1 cells. (**A–B**) Real-time RT-PCR detection of integrin β4 expression in (A) control (ctrl) and netrin-1-over-expressing (ntn1+) AsPC-1 cells and in (B) AsPC-1 cells treated with recombinant netrin-1 protein at the indicated concentrations. GAPDH was used as an internal control. (**C**) Western blotting analysis of integrin β4 expression level in control (ctrl) and netrin-1-over-expressing AsPC-1 cells (ntn1+; left panels) and in the AsPC-1 cells treated with the indicated concentration of recombinant netrin-1 protein (right panels). GAPDH was used as internal control.

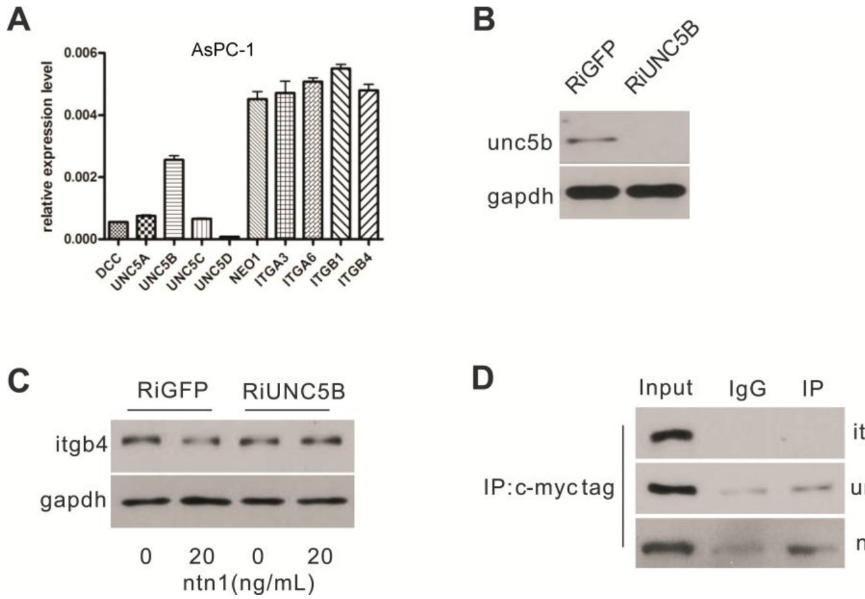

Supplementary Figure S4: Netrin-1 regulates integrin β4 expression through its receptor UNC5b. (**A**) Real-time PCR analysis for the expression of netrin-1 receptors in AsPC-1 cells. GAPDH was used as an internal control. (**B**) Western blotting analysis of UNC5b expression in the UNC5B RNAi and control GFP RNAi AsPC-1 cells. (**C**) Netrin-1 suppresses integrin β4 expression in the control GFP RNAi cells but not in the UNC5B RNAi cells. UNC5b knockdown and control AsPC-1 cells were treated with recombinant netrin-1, and the expression of integrin β4 before and after netrin-1 treatment was detected by western blotting. (**D**) Netrin-1 interacts with UNC5b but not integrin β4 in the netrin-1-over-expressing AsPC-1 cells. Immunoprecipitation was performed with an anti-myc antibody to pull-down the myc-tagged netrin in netrin-1-over-expressing AsPC-1 cells, followed by immunoblotting analysis of integrin β4 (itgb4), UNC5b and netrin-1 (ntn1) levels in the precipitate.

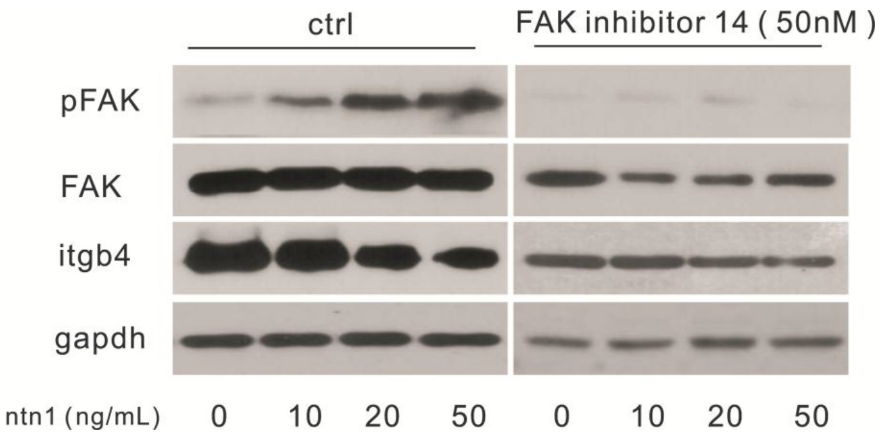

Supplementary Figure S5: Netrin-1 down-regulates integrin β4 expression by FAK activation. Western blotting assay of FAK, phospho-FAK (pFAK) and integrin β4 (itgb4) levels in AsPC-1 cells treated with the indicated concentrations of recombinant netrin-1 in the presence or absence of FAK inhibitor 14 (50 nM). GAPDH was shown as internal control. FAK inhibition abrogates the netrin-1-induced down-regulation of integrin β4 in AsPC-1 cells.

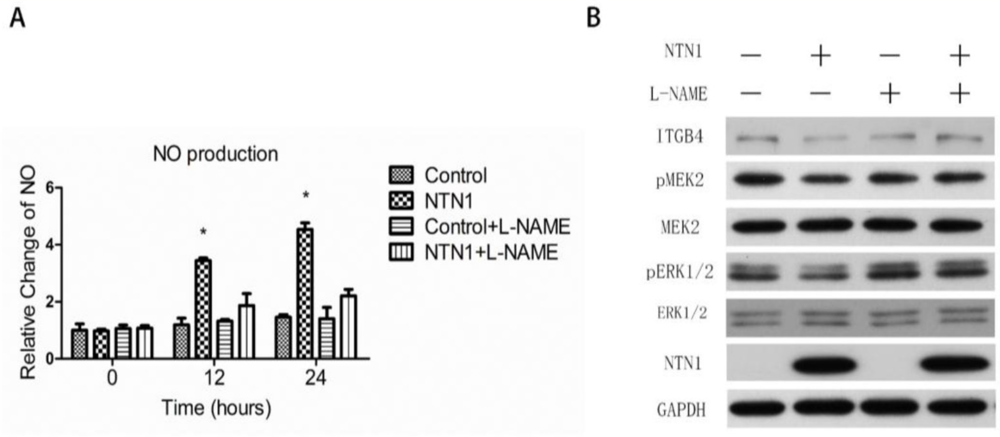

Supplementary Figure S6: NO production is necessary for Netrin-1 to induce suppression of MEK/ERK signaling and to inhibit integrin β4 expression. (**A**) Netrin-1 induces NO production in MiaPaCa II cells and its clearance by the treatment of NOS inhibitor L-NAME. (**B**) Netrin-1 induced suppression of MEK/ERK signaling and integrin β4 inhibition are abolished by L-NAME treatment.

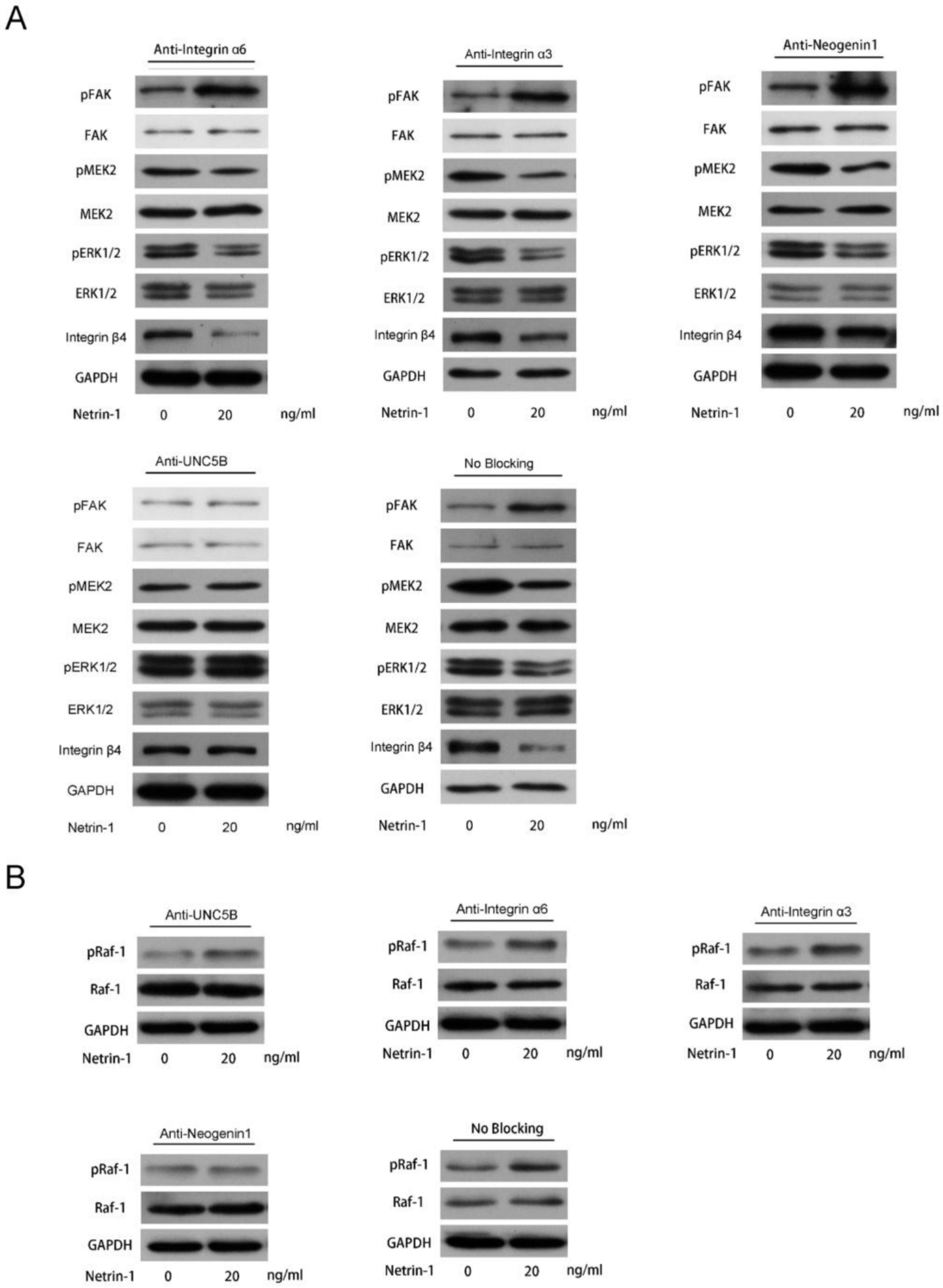

Supplementary Figure S7: UNC5B receptor mediates the inhibitory effect of netrin-1 to MEK/ERK signaling, while Neogenin1 receptor mediates the activation of c-RAF by netrin-1. Receptors on MiaPaCa II cells were blocked by their respective antibodies before treatment with netrin-1 (20 ng/ml). Protein level and phosphorylation of (**A**) FAK, MEK2, ERK1/2 and (**B**) c-Raf in the cells was then detected by western blotting. GAPDH was used as an internal control.

Supplementary Table S1: Interfering RNA sequences for ITGB4 and UNC5B

ITGB4	Forward strand	5′-gatccGCACGTGTGAGGAATGCAATTCAAGAGATTGCATTCCTCACACGTGCTTTTTTg-----3′
	Reverse strand	5′-aattcAAAAAAGCACGTGTGAGGAATGCAATCTCTTGAATTGCATTCCTCACACGTGCg-----3′
UNC5B	Forward strand	5′-gatccGCCACACAGATCTACTTCAATTCAAGAGATTGAAGTAGATCTGTGTGGTTTTTTg-----3′
	Reverse strand	5′-aattcAAAAAACCACACAGATCTACTTCAATCTCTTGAATTGAAGTAGATCTGTGTGGCg-----3′

Supplementary Table S2: Primers for real-time quantitative PCR

ITGB4 (NM_000213.3)	Forward primer	5′-GGGCAACATCCATCTGAAACC-3′
	Reverse primer	5′-CACTGTCCGCACACGAAGTCT-3′
ITGA6 (NM_000210.2)	Forward primer	5′-CCATTCCCATAACTGCCTCA-3′
	Reverse primer	5′-CGTCTCCACATCCCTCTTTT-3′
ITGA3 (NM_005501.2)	Forward primer	5′-CCCCAACTACAGGCGAAAC-3′
	Reverse primer	5′-AGAAGAAGCCGTGGAAGACA-3′
ITGB1 (NM_002211.3)	Forward primer	5′-CCTGCCTTGGTGTCTGTGC-3′
	Reverse primer	5′-GGGTAATTTGTCCCGACTTTCTA-3′
GAPDH (NM_002046.5)	Forward primer	5′-GGTCACCAGGGCTGCTTTTA-3′
	Reverse primer	5′-GAGGGATCTCGCTCCTGGA-3′
DCC (NM_005215.3)	Forward primer	5′-AGCCGATTTGTCCGTCTCA-3′
	Reverse primer	5′-TGTTGTATTCAATGCTCGTTCC-3′
NEO1 (NM_001172623.1)	Forward primer	5′-AAAGAGGGGAAACCTAAGACCA-3′
	Reverse primer	5′-CCCACAACAGGCTCAATAACC-3′
UNC5A (NM_133369.2)	Forward primer	5′-TGGCTGACTCGTCCATTCTC-3′
	Reverse primer	5′-GTGGTGGTGGTGGTGCTGA-3′
UNC5B (NM_170744.4)	Forward primer	5′-GTCCTTGAACATTAGCGGTTT-3′
	Reverse primer	5′-GACACGCCTGTAGCACTGAA-3′
UNC5C (NM_003728.3)	Forward primer	5′-GCGGACTGGGACTGGGATA-3′
	Reverse primer	5′-GTGGCAGAGGCTCAGGTGG-3′
UNC5D (NM_080872.2)	Forward primer	5′-CAAGGACTGGCAGATGTTAGC-3′
	Reverse primer	5′-TTCAAAATGACAGCAGATGGG-3′
